# A Wearable Molecularly Imprinted Electrochemical Sensor for Cortisol Stable Monitoring in Sweat

**DOI:** 10.3390/bios15030194

**Published:** 2025-03-18

**Authors:** Yitao Chen, Zidong He, Yuanzhao Wu, Xinyu Bai, Yuancheng Li, Weiwei Yang, Yiwei Liu, Run-Wei Li

**Affiliations:** 1 School of Materials Science and Chemical Engineering, Ningbo University, Ningbo 315000, China; chenyitao@nimte.ac.cn (Y.C.); baixy@nimte.ac.cn (X.B.); liyuancheng@nimte.ac.cn (Y.L.); 2 Ningbo Institute of Materials Technology and Engineering, Chinese Academy of Sciences, Ningbo 315000, China; hezidong@nimte.ac.cn (Z.H.); liuyw@nimte.ac.cn (Y.L.); 3 State Key Laboratory of Urban Water Resource and Environment, School of Chemistry and Chemical Engineering, Harbin Institute of Technology, Harbin 150000, China; yangww@hit.edu.cn

**Keywords:** mental stress, sweat cortisol, molecularly imprinted polymer, electrochemical sensor, liquid metal

## Abstract

Cortisol, a steroid hormone, is closely associated with human mental stress. The rapid, real-time, and continuous detection of cortisol using wearable devices offers a promising approach for individual mental health. These devices must exhibit high sensitivity and long-term stability to ensure reliable performance. This study developed a wearable electrochemical sensor based on molecularly imprinted polymer (MIP) technology for real-time and dynamic monitoring of cortisol in sweat. A flexible gold (Au) electrode with interfacial hydrophilic treatment was employed to construct a highly stable electrode. The integration of a silk fibroin/polyvinylidene fluoride (SF/PVDF) composite membrane facilitates directional sweat transport, while liquid metal bonding enhances electrode flexibility and mechanical anti-delamination capability. The sensor exhibits an ultrawide detection range (0.1 pM to 5 μM), high selectivity (over 100-fold against interferents such as glucose and lactic acid), and long-term stability (less than 3.76% signal attenuation over 120 cycles). Additionally, a gradient modulus design was implemented to mitigate mechanical deformation interference under wearable conditions. As a flexible wearable device for cortisol monitoring in human sweat, the sensor’s response closely aligns with the diurnal cortisol rhythm, offering a highly sensitive and interference-resistant wearable solution for mental health monitoring and advancing personalized dynamic assessment of stress-related disorders.

## 1. Introduction

In recent years, the COVID-19 pandemic [1] has caused a 25% global increase in the prevalence of anxiety and depression [2]. Chronically elevated stress levels not only weaken the immune system [3] but also contribute to diseases such as hypertension, cardiovascular disorders, and post-traumatic stress disorder [4,5,6,7]. Under excessive stress, the hypothalamic–pituitary–adrenal (HPA) axis [8] in the brain is activated, triggering the release of biomarkers, including adrenaline and cortisol [9,10] into body fluids. Cortisol, a key biomarker, is present in various body fluids, including sweat, saliva, urine, and blood (serum) [11,12]. Due to its noninvasive and easily collectible nature, sweat is the preferred sample for monitoring [13]. Therefore, real-time monitoring of sweat cortisol levels holds significant potential for both personal stress management and clinical diagnosis. 

Several techniques have been developed for detecting sweat cortisol in the medical field, including high-performance liquid chromatography-mass spectrometry (HPLC-MS) [14], enzyme-linked immunosorbent assay (ELISA) [15], surface plasmon resonance (SPR) [16], and electrochemiluminescence (ECL) [17]. Although these methods offer high sensitivity and accuracy, they are constrained by high costs, bulky instrumentation, specialized operational requirements, and lengthy detection procedures, rendering them unsuitable for continuous real-time monitoring [18,19,20]. While electrochemical (EC) sensors based on antibodies, enzymes, or aptamers [21,22,23] exhibit excellent selectivity [24], their stability is highly susceptible to environmental factors, which limits their reusability [25]. Researchers have investigated molecularly imprinted polymer (MIP) technology [26] as a potential solution to these challenges. MIP is a bionic artificial material [27] that selectively attaches to target particles based on template molecular recognition properties, which is widely used in the field of EC sensing. In the case of cortisol as a template molecule, MIP can be used as a specific recognition element for sensors to selectively detect or extract cortisol from complex biological fluids [28]. MIP is synthesized through the copolymerization of a monomer with the cortisol template molecule. After the template is removed, a specific recognition cavity [29] is formed. The concentration of the target molecule is then detected based on changes in the electrochemical signal when the target molecule reenters the cavity. EC sensors based on MIP technology are ideal for detecting cortisol in sweat. Sanjida et al. [30] significantly enhanced the sensitivity of the molecularly imprinted polymer (MIP) sensor through in situ synthesis of gold nanoparticles on the glassy carbon electrode, achieving ultrasensitive sub-picomolar cortisol detection. This approach leverages the localized surface plasmon resonance and catalytic activity of AuNPs to amplify electrochemical signals. Mei et al. [31] proposed a wearable electrochemical sensor integrating microfluidics and molecularly imprinted polymers (MIPs) for real-time cortisol monitoring in sweat, demonstrating several groundbreaking advantages in the field of personalized health monitoring. The above work achieves high-sensitivity detection of cortisol and provides new design ideas for wearable EC sweat sensors. However, current research exhibits significant limitations in assessing the operational stability of wearable devices under dynamic conditions, particularly with respect to mechanical stress cycles caused by body movements and the long-term performance of sensors in complex physiological environments.

**Figure 1:**
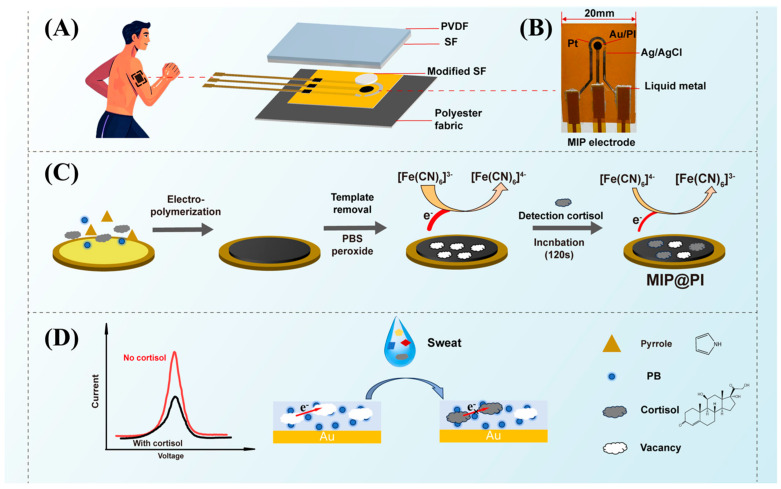
MIP@PI device, molecular imprinting electrode preparation process, and schematic of specific recognition detection of cortisol in sweat. (**A**) Schematic diagram of the wearable sensor tested on the skin surface and the structural schematic diagram of the device split, (**B**) Physical diagram of the MIP electrode, (**C**) Flowchart of the MIP electrode preparation process, (**D**) Schematic diagram of the sensor’s specific recognition of cortisol in sweat(The illustration shows the substances represented by each symbol).

This work developed a wearable molecularly imprinted electrochemical sensor for stable cortisol monitoring in sweat. As shown in Figure 1A, the electrochemical electrode was fabricated by depositing gold (Au) metal electrodes onto a polyimide (PI) flexible substrate, followed by electrochemical hydrophilic activation, optimization of electrode deposition and electropolymerization processes significantly enhances MIP electrode performance. Sweat was automatically collected and directionally transported by leveraging the surface hydrophilicity and hydrophobicity differences between an electrospun silk membrane and a polyvinylidene fluoride (PVDF) membrane (Figure 1A,B is the actual image of the electrode, and Figure S1 is the thickness of the device). When the device interfaces with sweat, the composite film efficiently directs the sweat flow toward the MIP sensing electrode (Figure 1C). The molecularly imprinted cavities selectively capture cortisol molecules, which subsequently impede the electron transfer of Prussian blue (PB). The cortisol concentration is then quantified by analyzing the corresponding changes in the electrochemical signal (Figure 1D). The optimized MIP sensor exhibited precise cortisol detection within a concentration range of 0.1 pM to 5 μM and maintained stable performance over 120 cycles, with only a slight decline in detection capability. Additionally, the sensor demonstrated high specificity for cortisol, even in the presence of other sweat components such as glucose, lactic acid, uric acid, and urea. Furthermore, a gradient modulus design was introduced, incorporating polyester fabric as an encapsulation layer to effectively dissipate strain generated under wearable conditions, thereby improving durability and usability. As a proof of concept, the sensor was attached to the arms of healthy volunteers to monitor sweat cortisol levels during different exercise stages. The results revealed the diurnal rhythm [32] of cortisol release. This approach provides a novel and effective method for cortisol monitoring and mental stress management. 

## 2. Materials and Methods

### 2.1. Materials 

The chemicals and materials used in this study included: Polyimide solutions (Purchased from Dongguan Chenyang Polymer Material Co., Ltd., Dongguan, China), fibroin (Purchased from Shiquan County laowantong Shannan specialty shop, Ankang, China), cortisol (Purchased from Chengdu Sitiande Biotechnology Co., Ltd., Chengdu, China), Potassium ferricyanide, hydrochloric acid, sulfuric acid, lactic acid (Purchased from Sinopharm Chemical Reagent Co., Ltd., Shanghai, China). Pyrrole, Potassium ferricyanide solution (PBS) buffer (pH = 7.4), urea, DMF, P(VDF-HFP) (Purchased from Shanghai McLean Biochemical Technology Co., Ltd., Shanghai, China). Formic acid, glucose, acetic acid, methanol, ethanol, sodium hydroxide, sodium chloride, ferric chloride, potassium chloride, polyethylene oxide, gallium indium alloy (Purchased from Shanghai Aladdin Biochemical Technology Co., Ltd., Shanghai, China). Deionized water, uric acid (Purchased from Shanghai Meryer Biochemical Technology Co., Ltd., Shanghai, China). Commercial platinum counter electrode, commercial silver/silver chloride reference electrode (Purchased from Tianjin AIDA Hengsheng Technology Development Co., Ltd., Tianjin, China). Flexible flat cable (FPC) (Purchased from Ruixing FPC flexible board manufacturer, Shenzhen, China). The specific information is in Table S1.

### 2.2. Preparation of the Electrochemical Three-Electrode System

Firstly, the Au working electrode and conductive circuit were deposited on a polyimide (PI) film via electron beam thermal evaporation. Subsequently, a Pt counter electrode was deposited on the conductive circuit using magnetron sputtering, followed by electron beam thermal evaporation of Ag as the base material for the Ag/AgCl reference electrode (all deposition processes employed customized mechanical masks to ensure precise electrode geometry and dimensions). The Ag/AgCl reference electrode was then electrochemically chlorinated using cyclic voltammetry (CV) [33] in a 0.1 M NaCl solution, with a potential range of −0.2 V to 0.7 V, 10 cycles, and a scan rate of 0.05 V/s. All three electrodes were connected via an FPC flexible flat cable, with liquid gallium–indium alloy applied at the junctions as an adhesive.

Secondly, the Au working electrode was activated in 0.1 M NaOH solution by (CV) within a potential range of −0.4 V to 1.5 V at a scan rate of 0.1 V/s for 10 cycles, ensuring the activation and cleaning of the electrode surface. Immediately after activation, the electrode was rinsed with deionized water and dried under a nitrogen stream. A (pyrrole-prussian blue)Py-PB oxide film was deposited onto the activated Au working electrode via electropolymerization in a solution containing 50 mM pyrrole, 50 mM cortisol, 5 mM potassium ferricyanide, and 5 mM ferric chloride in 0.05 M PBS buffer (pH = 7.4). Electropolymerization was conducted under the following conditions: a potential range of 0.4 V to 1.4 V, and a scan rate of 0.05 V/s, and 20 cycles (Figure S2A). Magnetic stirring was applied during the electropolymerization process to ensure adequate reaction of potassium ferricyanide with ferric chloride. The resulting molecularly imprinted polymer (MIP) membrane was rinsed with deionized water and dried under a nitrogen stream.

During this process, cortisol acts as a template molecule that interacts with the polymer network, while PB, which acts as a redox probe, co-polymerizes with pyrrole to form a Py-PB oxide film that is electro-polymerized onto the Au working electrode surface (Figure 1B). Throughout polymerization, the target molecules diffuse toward the Au electrode surface and interact with the Py matrix via hydrogen bonding. After 20 CV cycles, a uniform and dense Py-PB layer [34] with nanosized spherical particles was deposited onto the Au electrode surface.

Following electropolymerization, the polymer composite membrane containing the cortisol template was oxidized in 0.05 M PBS buffer (pH 7.4) within a potential range of 0.4 V to 1.7 V for 20 cycles at a scan rate of 0.05 V/s to remove the cortisol template molecules (Figure S2B). During this step, the oxidation process weakens the hydrogen bond between cortisol and the Py matrix, facilitating the effective elution of cortisol and the formation of specific recognition cavities in the polymer. Additionally, a non-imprinted polymer (NIP) was prepared under identical conditions, except that the electropolymerization solution did not contain cortisol. This control experiment was conducted to further confirm the specificity and functionality of the MIP.

### 2.3. Preparation of Silk/PVDF Composite Film

A silk/PVDF composite film with sweat directional transport function was prepared by electrospinning technology. Firstly, the silk spinning solution was prepared by dissolving silk fibroin (15 wt%) in formic acid (85%) with the addition of polyethylene oxide (PEO, 2 wt% of silk fibroin). The mixture was stirred for 10 h until the silk fibroin was completely dissolved. The resulting solution was then loaded into a 5 mL syringe for electrospinning. The electrospinning parameters were set as follows: needle type 23G, temperature 25 °C, humidity 40%, voltage 1.4 kV/cm, needle-to-collector distance of 10 cm, and injection speed of 0.16 mm/min. After electrospinning, the silk film was soaked in glycerol for 5 h and subsequently dried in an oven at 60 °C for 2 h. The PVDF spinning solution was prepared by mixing DMF and P(VDF-HFP) in an oil bath at 70 °C with a mass ratio of 4:1 and stirring at 600 rpm for 6 h. The PVDF spinning solution was then loaded into a 5 mL syringe for electrospinning. To fabricate the composite film, the silk film was first fixed onto the collector. The electrospinning parameters were as follows: needle type 21G, temperature 25 °C, humidity 40%, voltage 1.4 kV/cm, needle-to-collector distance of 10 cm, and injection speed of 0.3 mm/min.

### 2.4. Fabrication of MIP Electrochemical Sensor

The MIP@PI sensor was fabricated on a flexible polyimide (PI) substrate and integrated with a directional sweat transport layer composed of a silk/PVDF composite membrane. This membrane, positioned over the electrode array, enables controlled sweat flow toward the sensing area. The device was encapsulated by sandwiching the sensor between the silk/PVDF layer and a breathable polyester fabric, bonded via hot-melt adhesive. Hot-pressing at 100 °C for 120 s ensured robust interlayer adhesion while preserving the membrane’s microfluidic functionality and mechanical flexibility.

### 2.5. Characterization and Detection

Electrode deposition was carried out using electron beam thermal evaporation and magnetron sputtering [35]. Electrochemical characterizations, including cyclic voltammetry (CV), chronoamperometry (CA), and differential pulse voltammetry (DPV), were conducted using a Zahner Electrochemical Workstation. The morphology of the molecularly imprinted polymer (MIP) electrode was analyzed by scanning electron microscopy (SEM) and energy-dispersive X-ray spectroscopy (EDS).

To characterize the performance of the sensor, the MIP sensor was initially incubated in 0.05M PBS buffer (pH 7.4) for 120 s. Immediately after incubation, the electrodes were flushed with deionized water and vacuum-dried. CV tests were conducted on all electrodes at room temperature in a 0.05M PBS with a potential range of −0.2 V to 0.8 V, scanned three times at a scanning rate of 0.05 V/s. The current response of the sensor before and after parameter optimization was measured using CA (fixed potentials under CA testing detect the strongest signals and reduce interference with results from other substances). The parameters were as follows: fixed potential of +1.08 V and scanning time of 120 s. Then, DPV was used to measure the detection range of the sensor after optimizing the parameters (the sensitivity of cortisol detection can be improved by pulse potential modulation, which allows the selection of a potential range independent of the chemical reaction). The parameters were as follows: potential range −0.2 V~0.6 V, pulse amplitude 50 mV/s, pulse time 10 ms, e-step length 4 mV, scanning rate 50 mV/s. 

## 3. Results and Discussion

### 3.1. Characterization of the Electrochemical Three-Electrode System

**Figure 2:**
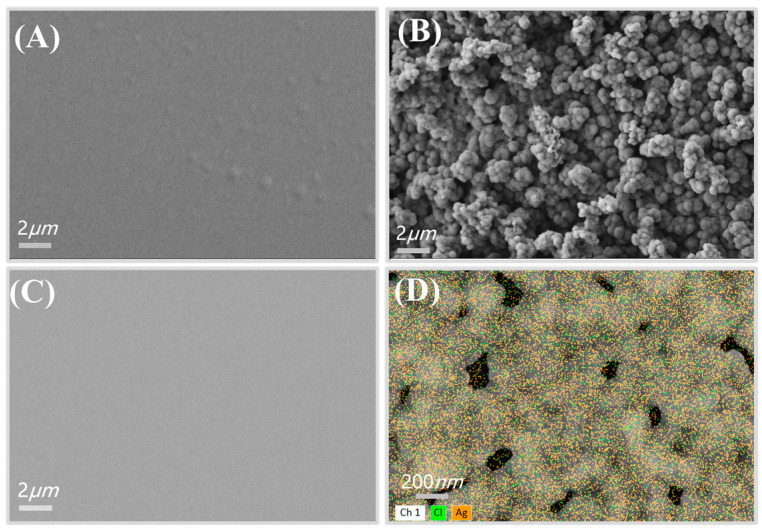
SEM characterization of electrode morphology, (**A**) activated Au working electrode, (**B**) deposited PB-Py molecular-imprinted oxide layer on Au electrode, (**C**) Pt electrode, (**D**) Ag/AgCl reference electrode EDS spectra.

The surface morphology of each electrode in the MIP sensor was characterized using scanning electron microscopy (SEM) and energy-dispersive X-ray spectroscopy (EDS). Prior to the electropolymerization of the Py-PB composite membrane, the SEM image (Figure 2A) reveals a rough gold (Au) electrode surface following activation. This surface roughness increases the number of binding sites for Py-PB particles, facilitating their adsorption and promoting the efficient transfer of the polymer matrix during electropolymerization. After electropolymerization, the gold electrode surface is uniformly coated with a dense Py-PB layer composed of nanoscale spherical particles (Figure 2B). The SEM image of the platinum (Pt) electrode demonstrates a uniformly distributed, continuous conductive structure, which enhances sensor stability due to its robust and smooth surface (Figure 2C). The EDS spectrum (Figure 2D) shows the cross-distribution of silver (Ag) and chlorine (Cl) elements, confirming the successful fabrication of the Ag/AgCl reference electrode via the cyclic voltammetry (CV) method. Additionally, the SEM images in Figure S3A illustrate a smooth, crack-free silver (Ag) electrode prior to chlorination (Figure S3B), as well as the chlorinated reference electrode. 

### 3.2. Parameter Optimization of Membrane Process of Py-PB Films

**Figure 3:**
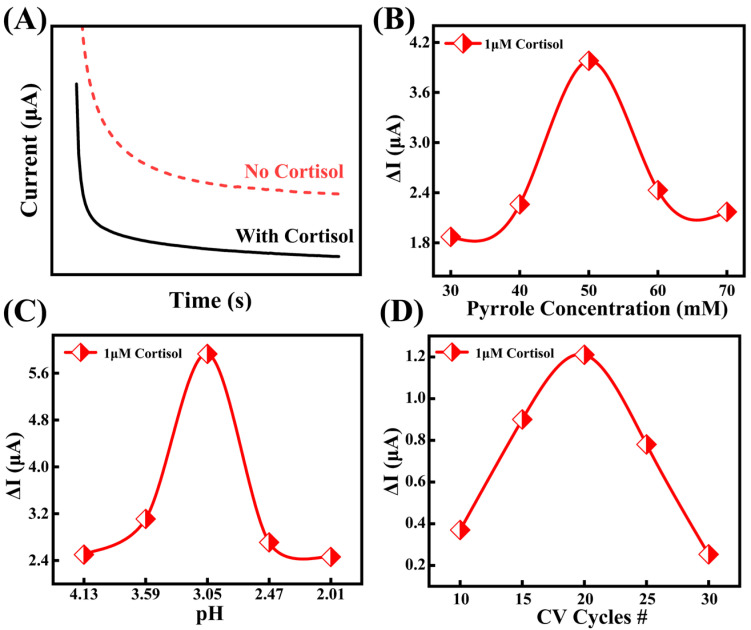
Optimization of preparation parameters, (**A**) chronoamperometric measurement of MIP electrode, (**B**) effect of pyrrole concentration on electrode sensing, (**C**) effect law of solution pH on electrode sensing, (**D**) effect law of circulation number on electrode sensing.

To enhance the cortisol recognition capability and detection stability of the biosensor, we systematically studied and optimized the electropolymerization parameters for the in situ synthesis of Py-PB film on an activated bare Au electrode, which functions as the working electrode in the sensor. Upon contact with cortisol, the recognition sites within the MIP layer are occupied by cortisol molecules, thereby impeding the electron transfer pathway of PB [36]. To quantify this effect, a calibration curve was established by correlating cortisol concentration with the current response obtained from chronoamperometry (CA) (Figure 3A). Specifically, CA tests were conducted to evaluate the current response of electrodes fabricated under varying electropolymerization parameters across different cortisol concentrations. The maximum current (*I*_M_) and saturation current (*I*_S_) were recorded, and the current difference (Δ*I* = *I*_M_ − *I*_S_) was calculated. Δ*I* values were then compared to determine the optimal process parameters. Throughout all experiments, the electropolymerization scan rate and cyclic voltammetry (CV) potential range remained constant. Consistent with previous studies, the scanning rate was set at 0.05 V/s, and the CV potential range was fixed between 0.4 and 1.4 V. Other parameters were initially set as follows: monomer concentration at 40 mM, number of cycles at 10, polymerization solution pH at 4.96, and 0.1M NaOH solution was used for cortisol elution. A controlled variable method was applied to optimize all parameters individually.

Firstly, the effect of pyrrole monomer concentration on the MIP electrode was investigated. The cortisol template concentration was fixed at 50 mM, while varying pyrrole concentrations (30, 40, 50, 60, and 70 mM) were used for MIP electrode preparation. The influence of Py concentration was evaluated by measuring the CA current response of the MIP electrode after template removal in a 1 μM cortisol solution. As shown in Figure 3B, when the pyrrole monomer concentration was below 50 mM, the *ΔI* value of the current response in 1 μM cortisol solution reached 1.89 μA. This relatively low current response could be attributed to the insufficient monomer concentration during electropolymerization, which led to a reduced number of cortisol template molecules binding to the monomer. Consequently, after template removal, fewer recognition cavities were formed, leading to a weaker current difference when cortisol molecules were detected again. However, when the pyrrole monomer concentration exceeded 50 mM, the Δ*I* value in the 1 μM cortisol solution exhibited a downward trend. This was attributed to the non-conductive nature of the Py-PB layer formed during electropolymerization [37]. A higher monomer concentration led to the formation of an excessively thick oxide layer, which hindered electron conductivity. As a result, after template removal, the cavities became less responsive to cortisol molecules, leading to a smaller difference in current response. In summary, to achieve an optimal balance between current response and structural stability, and to obtain well-defined and stable recognition cavities, 50 mM was determined to be the most suitable pyrrole monomer concentration. Based on this optimized condition, we further investigated the effect of CV cycle number on electrode performance.

To further optimize the effect of the polymerization solution’s pH on the MIP membrane polymerization process, the pH was adjusted by adding a diluted hydrochloric acid solution. As shown in Figure 3C, as the pH value gradually decreases, the current response Δ*I* of the MIP sensor increases, reaching a maximum of 5.93 μA. This indicates that a lower pH facilitates the oxidation of the pyrrole monomer during the electropolymerization process [38], thereby promoting the formation of the Py-PB oxide layer and generating more specific recognition cavities after eluting the cortisol template molecules. However, when the pH drops below 3.05, Δ*I* decreases to 2.7 μA. One reason for this is that excessively low pH affects the ionization state of the polymerization reaction, thereby interfering with the reaction rate. Additionally, the reduced current difference makes it difficult to distinguish between varying concentrations, compromising the sensor’s detection accuracy. Therefore, a pH of 3.05 was determined to be optimal, as it ensures efficient oxidation of the pyrrole monomer while avoiding the negative effects of excessive pH reduction on the sensor’s performance.

The number of CV cycles directly influences the degree of redox reaction and the thickness of the Py-PB oxide layer. As shown in Figure S4, the oxide layer thickness increases with the number of cycles. When the number of cycles reaches 20, the corresponding oxide layer thickness is 9 μm. At this thickness, the Δ*I* value of the current response in 1 μM cortisol solution peaks at 3.98 μA (Figure 3D). If the oxide layer is too thin, the cavity structure formed after template removal is weak [39], resulting in an insignificant difference between the maximum current and the saturation current. Conversely, if the oxide layer is too thick, as previously mentioned, it can hinder electron conductivity, resulting in a reduced current response difference. Therefore, 20 cycles is determined to be the optimal number of CV cycles.

### 3.3. Optimization of Template Removal Parameter and Incubation Time

After the electropolymerization of the MIP layer, cortisol template molecules were extracted from the polymer matrix, leaving specific recognition cavities that match the shape and size of the template molecules. The effective extraction of template molecules is crucial for the sensor’s detection and recognition capabilities [40]. Therefore, the extraction solution and method were optimized. Commonly used solvents include sodium hydroxide, ethanol, acetic acid, and a methanol/acetic acid (9:1, *v*/*v*) mixture. 

**Figure 4:**
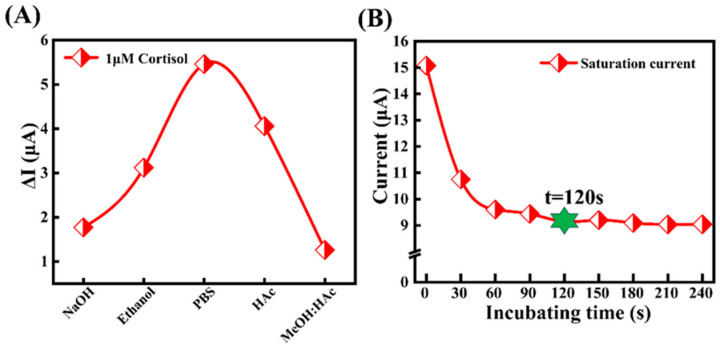
Optimization of template removal mode and influence of incubation electrode, (**A**) effects of different eluting solvents on electrode sensing performance, (**B**) effects of incubation time on electrode sensing.

To optimize the detection performance of the MIP sensor, different solvent extraction methods and the peroxidation technique were systematically evaluated (Figure 4A). As shown in the figure, the CV peroxidation method using a 0.05M PBS solution to remove cortisol template molecules exhibited the highest efficiency, with a response current (Δ*I*) of 5.461 μA. The extraction methods using 10% ethanol and 10% acetic acid solution also yielded good results, with the extraction efficiency ranking as follows: 10% ethanol > 10% acetic acid > 0.1 mol/L NaOH > methanol: acetic acid (9:1, *v*/*v*). Therefore, we selected the peroxidation technique as the optimal template removal method. Additionally, due to the superior extraction ability of 10% ethanol for cortisol, it was chosen as the extraction solvent for subsequent reuse.

After systematically optimizing the electropolymerization and template removal parameters, this study further investigated the interaction between cortisol and the MIP sensor [41]. In the experiment, the MIP sensor was first incubated in 0.05 mol/L PBS buffer for varying durations. The current response was then measured in a 1 μM cortisol solution using chronoamperometry. The experimental results show that as the incubation time increased, the current response decreased to 9.15 μA, eventually reaching a stable value at 120 s (Figure 4B). Therefore, to balance experimental efficiency and sensor response, 120 s was determined to be the optimal incubation time for subsequent experiments.

### 3.4. The MIP Sensor for Cortisol Detection 

**Figure 5:**
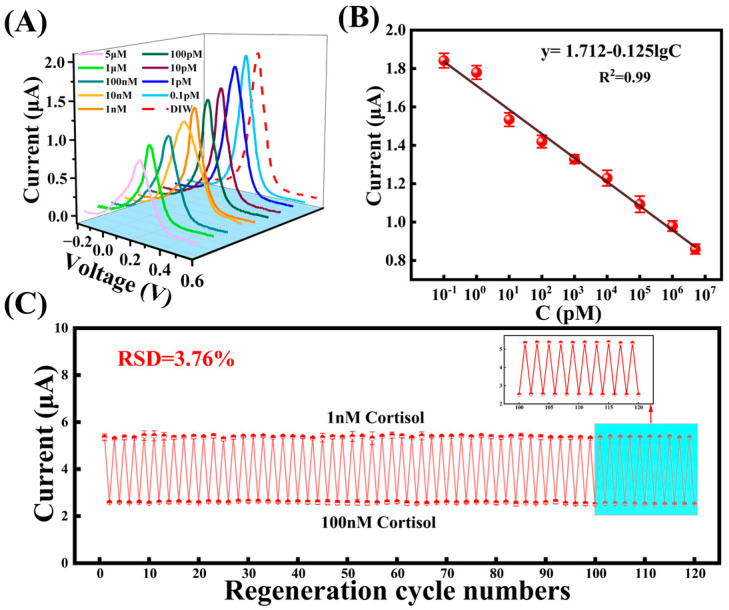
The sensor detects cortisol, (**A**) DPV current response of the sensor under different cortisol concentrations (0.1 pM–5 μM), (**B**) The sensor response fits the curve, (**C**) The sensor detects the current response of 120 regeneration cycles of 1 nM and 100 nM cortisol.

The peak current of the DPV signal for the MIP sensor bound to cortisol was recorded across a concentration range of 0.1 pM to 5 μM (Figure 5A). As the cortisol concentration increased, the peak current also increased proportionally. To quantitatively analyze the relationship between cortisol concentration and current, the DPV signal for each concentration was measured three times, and the linear regression equation was fitted as follows (Figure 5B):

y = 1.712 − 0.125LgC_cortisol_, (1)

where the C_cortisol_ is the concentration of cortisol, and R^2^ is 0.99. As a result, the MIP sensor exhibits excellent response sensitivity in the cortisol concentration range of 0.1 pM~5 μM.

In addition, the reproducibility of the MIP sensor was evaluated by repeatedly testing the sensor in 1nM and 100nM cortisol solutions (Figure 5C). At the end of each test cycle, the electrodes were soaked in anhydrous ethanol for 2 minutes to extract the cortisol molecules, followed by blowing the electrodes dry before the next test cycle. The results demonstrated that the MIP sensor successfully detected cortisol at both low (1 nM) and high (100 nM) concentrations through regeneration, with the regeneration cycle reaching up to 120 cycles (This regenerative approach was validated in the work of Hu et al. [34]). The relative standard deviation (RSD) of the last 20 cycles was 3.76%, indicating that the sensor maintained excellent stability during long-term continuous monitoring. Compared with flexible molecular imprinting sensors reported in recent years, this sensor exhibited the widest detection range and the lowest detection limit (Figure S5 and Table S2).

**Figure 6:**
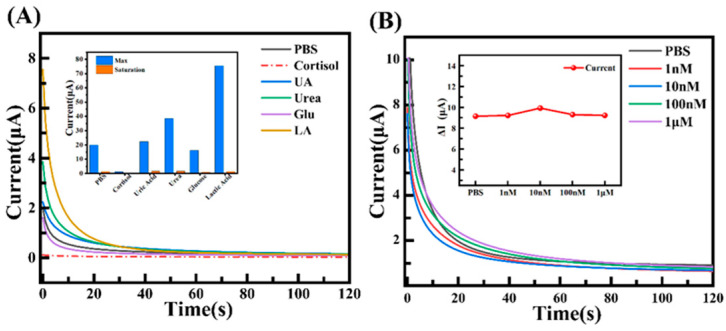
Specific selective characterization, (**A**) current response of MIP sensor under different substances, and (**B**) current response of NIP sensor under different concentrations of cortisol solution.

In order to verify the specific selectivity of the MIP sensor, the same fabrication process was used to prepare the sensor. The response of the sensor to common interfering substances commonly found at high concentrations in sweat was then tested. These substances included 5 mM urea, 5 mM lactic acid, 50 μM glucose, and 50 μM uric acid. As demonstrated in Figure 6A, the sensor exhibited a response current of 0.358 μA for 50 μM cortisol, while the current values for the other interfering substances were considerably higher. This outcome can be attributed to the elution process, which removes the template molecules, thereby forming cavities with geometric and chemical complementarity. In conjunction with the oriented functional groups of pyrroles, this process ensures high specificity recognition of cortisol. This mechanism endows the MIP sensor with the capability to accurately detect target molecules even in complex biological matrices. In contrast, the NIP sensor, which lacks specific imprinting cavities for cortisol (Figure S6), exhibited no significant differences in current response across cortisol solutions at varying concentrations (Figure 6B), thereby further confirming the imprinting effect of MIP technology. These findings underscore the potential of MIP sensors for the selective detection of specific biomarkers, particularly in complex biological matrices.

### 3.5. Performance Characterization of MIP Electrode and Sensor Stability Test

**Figure 7:**
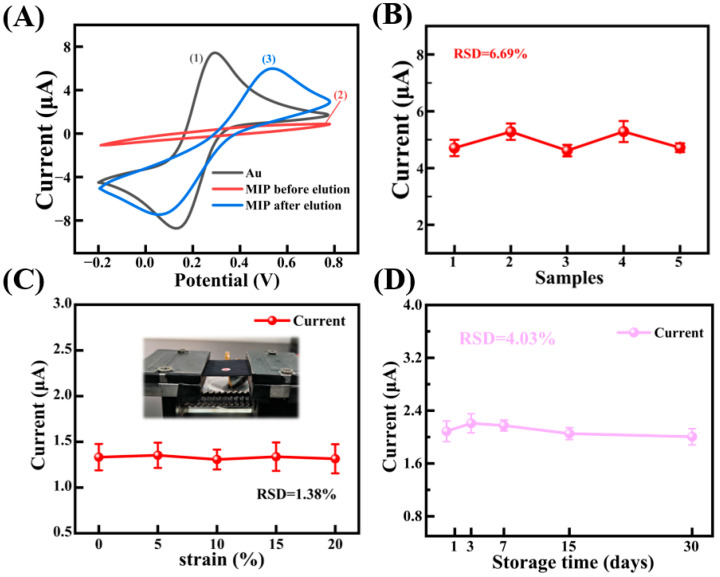
CV curves of electrodes in different stages and repeatability characterization of preparation process and sensor stability characterization, (**A**) CV curves at different stages of preparation of MIP electrode, (**B**) reproducibility of sensor, (**C**) current response of the device under strain state, (**D**) current response of the sensor within 30 days.

After optimizing all preparation parameters, we tracked the electrochemical behavior of the MIP electrode at different stages using the CV method (Figure 7A). Compared to the bare gold electrode (curve 1), the current signal was significantly reduced after the electropolymerization of the Py-PB layer (curve 2), indicating that the formation of the non-conductive layer hinders electron transfer, thereby suppressing the redox current of the electrode. After elution of the cortisol template (curve 3), the redox current increases significantly, demonstrating that the presence of cortisol disrupts the electron transfer path of PB. 

Additionally, to evaluate the reproducibility of the sensor fabrication process, five identical sensors were prepared using the same procedure. Each electrode was used to detect a 1 μM cortisol solution, as shown in Figure 7B. The maximum current response of each sensor was recorded using a chronoamperometric (CA) method, and the relative standard deviation (RSD) was calculated to assess the reproducibility of the process. The RSD obtained was 6.69%, indicating that the preparation process has high reproducibility.

To test the stability of the device, the MIP sensor was packaged with polyester cloth and a silk/PVDF composite film, then placed on a tensile fixture and subjected to five cycles of tensile strain at 20%. As shown in Figure 7C, under these five strain cycles, the CA current response of the sensor remained stable at 1.3 μA, with RSD of only 1.38% at 1 μM cortisol. This stability is attributed to the modulus mismatch between PI and polyester cloth (Figure S7). When the device is stretched, the deformation of the sensor center is minimized, improving the detection stability under strain [42]. For long-term stability testing, the device was stored at room temperature, and 1 μM cortisol was measured at intervals of 3 days, 7 days, half a month, and one month. As shown in Figure 7D, the decay in the response current was negligible, with 96.1% of the initial signal retained after 30 days, demonstrating the excellent long-term reliability of the proposed sensor. Additionally, the electrospun silk/PVDF membrane selectively transported cortisol-rich sweat while filtering out larger proteins or particulates. The polyester cloth barrier protected the sensor from environmental contaminants (e.g., dust, lipids). Gallium–indium alloy bonding improved electrode flexibility and resistance to delamination under mechanical stress.

### 3.6. Surface Sweat Test

**Figure 8:**
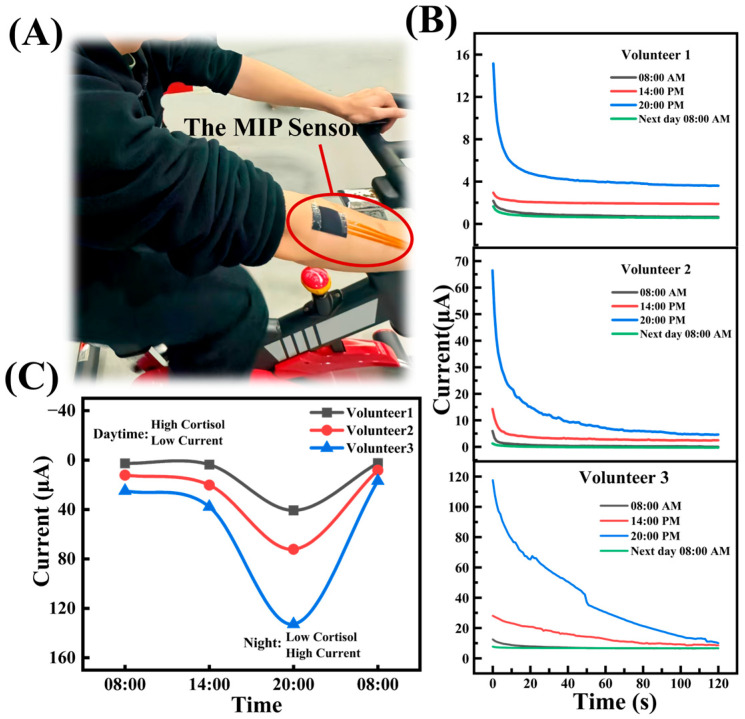
In vivo analysis of sweat on human body surface, (**A**) sensor attached to the subject’s arm during the exercise of volunteers on bicycles, (**B**) the CA current curves of three volunteers were tested at 08:00 AM, 14:00 PM, 20:00 PM and 08:00 am the next day, respectively, (**C**) circadian rhythm characterization.

To evaluate the wearable performance of the sensor, in vivo tests were conducted on three healthy volunteers over different periods. A medical pressure-sensitive adhesive was used to lightly attach the sensor to a volunteer’s arm while running on a treadmill (Figure 8A). And we collected samples every minute during volunteer exercise-induced sweating and measured the sensor’s response current (Figure S8). When cortisol molecules in sweat occupy the recognition sites on the MIP electrode, they obstruct the electron transfer pathway of PB, leading to a reduced CA current response within 150 s. Additionally, the polyester fabric material is soft, and its microtextured surface reduces contact area, which helps to minimize noise. As shown in Figure 8B, at 08:00 AM, the CA measurements of the three volunteers recorded the lowest current values: 3.03 μA, 7.47 μA, and 22.815 μA, respectively. However, the highest CA current values were observed at 8:00 PM, with measurements of 48.506 μA, 70.059 μA, and 134.53 μA, respectively. At 08:00 AM the following day, the CA current dropped again to the lowest values: 2.944 μA, 5.254 μA, and 15.3 μA, respectively. It can be found that the change in cortisol concentration in sweat follows the same pattern, reflecting the diurnal variation of cortisol secretion in human sweat (Figure 8C). 

## 4. Conclusions

In this study, we systematically investigated the key parameters involved in the fabrication of molecularly imprinted polymer (MIP) sensors for cortisol detection. Each parameter was carefully analyzed to establish a consistent and efficient fabrication process. Through the optimization of crucial parameters such as monomer concentration, cycle number, pH, template removal technique, and incubation conditions, we have developed a highly selective and reproducible MIP sensor capable of accurately detecting cortisol within a concentration range of 0.1 pM to 5 μM. Notably, the sensor demonstrated continuous and stable performance over 120 cycles without any degradation in sensing capability. This study proposes a precise and non-invasive strategy for cortisol detection, paving the way for applications in areas such as personal stress management and clinical diagnostics.

## Data Availability

All relevant data is included in the manuscript and in the Supplementary Materials.

## Supplementary Materials

The following supporting information can be downloaded at: https://www.mdpi.com/article/10.3390/bios15030194/s1, Figure S1. The thickness of the device. Figure S2. (A) CV curve of electropolymerization process, (B) Eluted cortisol CV curve. Figure S3. (A) Ag electrode, (B) Ag/AgCl reference electrode after chlorination by CV method. Figure S4. Relationship between CV Cycle Number and MIP Imprinted Layer Thickness. Figure S5. Compared with the performance of flexible molecularly imprinted sensors reported in recent years [30,31,34,38,41,43,44,45,46,47]. Figure S6. Surface morphology of NIP oxide layer. Figure S7. (A) Young’s modulus of polyester cloth and PI, (B) Stress distribution diagram of tensile simulation. Figure S8. Measure the sensor’s CA value every minute. Table S1. The suppliers, model numbers, brands, and suppliers of the materials used. Table S2. Comparison of sensor performance metrics with other reported works. Ref. [48] is cited in Supplementary Materials.
