# Supplementary material for: A Wearable Molecularly Imprinted Electrochemical Sensor for Cortisol Stable Monitoring in Sweat
Source: Biosensors (Basel). 2025 Mar 18;15(3):194. doi: 10.3390/bios15030194 (PMC11940103; doi:10.3390/bios15030194)
Supplement: Supplementary file 1 [file biosensors-15-00194-s001.zip › biosensors-3500564-supplementary.pdf]

Supplementary Material

A wearable molecularly imprinted electrochemical sensor for cortisol stable monitoring in sweat

Yitao Chen [1#], Zidong He [2#], Yuanzhao Wu [2*], Xinyu Bai[1], Yuancheng Li[1] , Weiwei Yang[3], Yiwei Liu[2] and Run-Wei Li [2*]

[1] School of Materials Science and Chemical Engineering, Ningbo University, Ningbo, China
[2] Ningbo Institute of Materials Technology and Engineering, Chinese Academy of Sciences, Ningbo, China
[3] State Key Laboratory of Urban Water Resource and Environment, School of Chemistry and Chemical Engineering, Harbin Institute of Technology, Harbin, China
[#]Co-author: Yitao Chen, Zidong He
*Correspondence: wuyz@nimte.ac.cn (Y.W.); runweili@nimte.ac.cn(R-W.Li)

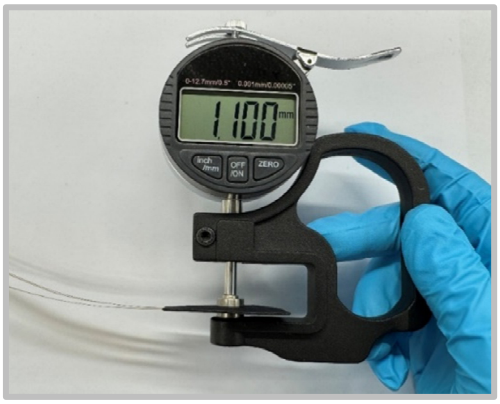

Figure S1. The thickness of the device.

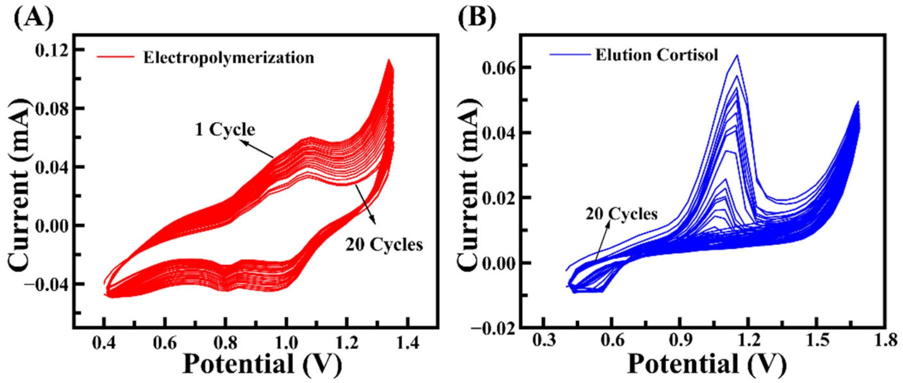

Figure S2. (A) CV curve of electropolymerization process, (B) Eluted cortisol CV curve.

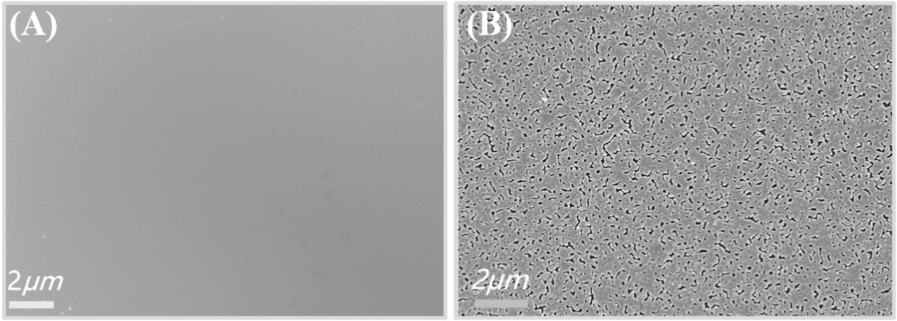

Figure S3. (A) Ag electrode,(B) Ag/AgCl reference electrode after chlorination by CV method.

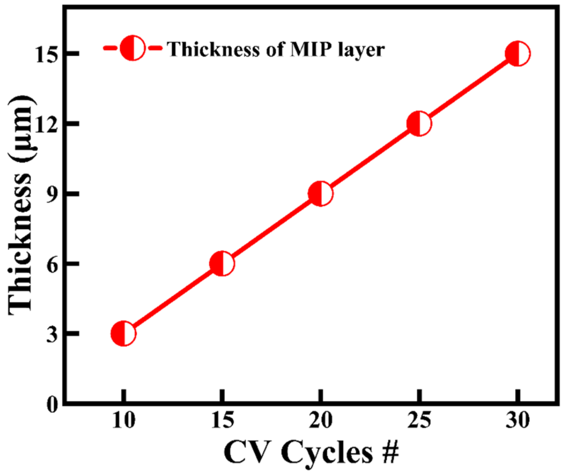

Figure S4. Relationship between CV Cycle Number and MIP Imprinted Layer Thickness.

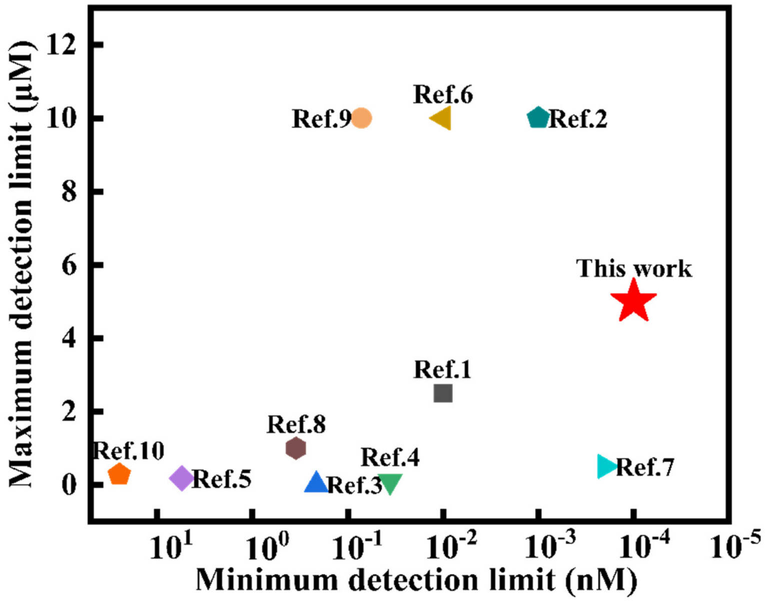

Figure S5. Compared with the performance of flexible molecularly imprinted sensors reported in recent years[1–10].

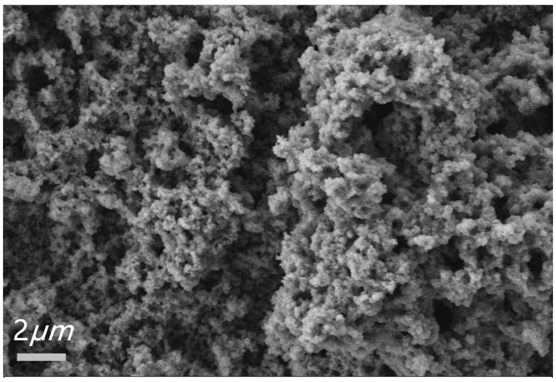

Figure S6. Surface morphology of NIP oxide layer.

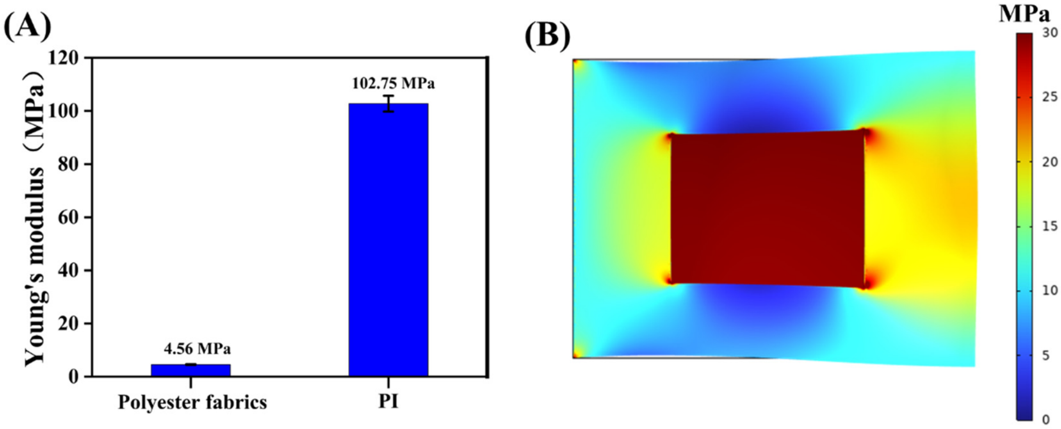

Figure S7. (A) Young's modulus of polyester cloth and PI, (B) Stress distribution diagram of tensile simulation.

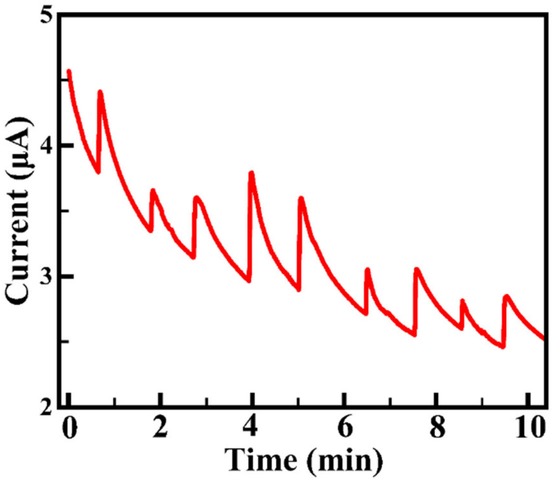

Figure S8. Measure the sensor's CA value every minute.

Table S1. The suppliers, model numbers, brands, and suppliers of the materials used.

Material	Vendor	Model	Make	Country
Polyimide solutions	Dongguan Chenyang Polymer Material Co., Ltd.	500ml	Dongguan Chenyang Polymer Material Co., Ltd.	China

Fibroin	Shiquan County laowantong Shannan specialty shop	200g	Shiquan County laowantong Shannan specialty shop
Cortisol	Chengdu Sitiande Biotechnology Co., Ltd.	10g	Chengdu Sitiande Biotechnology Co., Ltd.
Potassium ferricyanide	Sinopharm Chemical Reagent Co., Ltd	500g	Sinopharm Chemical
Hydrochloric acid		500ml	
Sulfuric acid		500ml	
Lactic acid		500ml	
Pyrrole	Shanghai Macklin Biochemical Technology Co., Ltd.	100ml	Macklin
PBS buffer		500ml	BBI
Urea		100g	Macklin
DMF		500ml	
P(VDF-HFP)	Shanghai Aladdin Biochemical Technology Co., Ltd.	500g	aladdin
Formic acid		500ml	
Glucose		500g	
Acetic acid		500ml	
Methanol		500ml	
Ethanol		5L	
Sodium hydroxide		500g	
Sodium chloride		500g	
Ferric chloride		200g	
Potassium chloride		500g	
Polyethylene oxide		500g	
Gallium indium alloy		5kg	
Deionized water	Shanghai Meryer Biochemical Technology Co., Ltd.	5L	Meryer
Uric acid		500ml	

Table S2. Comparison of sensor performance metrics with other reported works.

Materials	Sensitivity	Operation duration	Stability	Mechanical performances	Detection range	potential application areas	Ref
Au/SPE	2.0μA/nM	30min	15	Rigid	0.05nM-2.5μM	Saliva	[1]
CNT	0.048μA/nM	10min	100	Flexible	1pM-10μM	Sweat	[2]
AgNP/GE	4.695×10^{-6}μA/nM	-	10	Rigid	0.395nM-3.96nM	Plasma	[3]
PVA	0.038μA/nM	3.5min	50	Flexible	1pM-1μM	Sweat	[6]
GCE/AuNP	0.252μA/nM	8min	7	Rigid	1 pM-500 nM	Saliva	[7]
PET/CNT /AuNP	0.117μA/nM	21min	20	Flexible	1nM~1μM	Sweat	[8]
PET/C/Ag	2.3μA/nM	3min	1	Flexible	0.276nM-27.6μM	Sweat	[11]
PI/Au	0.125μA/nM	2min	120	Flexible	0.1pM-5μM	Sweat	This work

References

1. Karthika, P.; Shanmuganathan, S.; Subramanian, V.; Delerue-Matos, C. Selective Detection of Salivary Cortisol Using Screen-Printed Electrode Coated with Molecularly Imprinted Polymer. *Talanta* **2024**, *272*, 125823.
2. Hu, X.; Chen, Y.; Wang, X.; Jia, K.; Zhang, H.; Wang, Y.; Chu, H.; Zhong, X.; Lin, M.; Chen, P.; et al. Wearable and Regenerable

Electrochemical Fabric Sensing System Based on Molecularly Imprinted Polymers for Real-Time Stress Management. *Adv. Funct. Mater.* **2024**, *34*, 2312897.
3. Shama, N.A.; Aşır, S.; Göktürk, I.; Yılmaz, F.; Türkmen, D.; Denizli, A. Electrochemical Detection of Cortisol by Silver Nanoparticle-Modified Molecularly Imprinted Polymer-Coated Pencil Graphite Electrodes. *ACS Omega* **2023**, *8*, 29202–29212.
4. Kim, M.; Park, D.; Park, J.; Park, J. Bio-Inspired Molecularly Imprinted Polymer Electrochemical Sensor for Cortisol Detection Based on O-Phenylenediamine Optimization. *Biomimetics* **2023**, *8*, 282.
5. Liu, H.; Qin, W.; Li, X.; Feng, L.; Gu, C.; Chen, J.; Tian, Z.; Chen, J.; Yang, M.; Qiao, H.; et al. Molecularly Imprinted Electrochemical Sensors Based on $Ti_3C_2T_x$-MXene and Graphene Composite Modifications for Ultrasensitive Cortisol Detection. *Anal. Chem.* **2023**, *95*, 16079–16088.
6. Tang, W.; Yin, L.; Sempionatto, J.R.; Moon, J.; Teymourian, H.; Wang, J. Touch-Based Stressless Cortisol Sensing. *Adv. Mater.* **2021**, *33*, 2008465.
7. Yeasmin, S.; Wu, B.; Liu, Y.; Ullah, A.; Cheng, L.-J. Nano Gold-Doped Molecularly Imprinted Electrochemical Sensor for Rapid and Ultrasensitive Cortisol Detection. *Biosens. Bioelectron.* **2022**, *206*, 114142.
8. Mei, X.; Yang, J.; Yu, X.; Peng, Z.; Zhang, G.; Li, Y. Wearable Molecularly Imprinted Electrochemical Sensor with Integrated Nanofiber-Based Microfluidic Chip for in Situ Monitoring of Cortisol in Sweat. *Sens. Actuators B Chem.* **2023**, *381*, 133451.
9. Zhao, H.; Zhang, X.; Qin, Y.; Xia, Y.; Xu, X.; Sun, X.; Yu, D.; Mugo, S.M.; Wang, D.; Zhang, Q. An Integrated Wearable Sweat Sensing Patch for Passive Continuous Analysis of Stress Biomarkers at Rest. *Adv. Funct. Mater.* **2023**, *33*, 2212083.
10. Mugo, S.M.; Robertson, S.V.; Lu, W. A Molecularly Imprinted Screen-Printed Carbon Electrode for Electrochemical Epinephrine, Lactate, and Cortisol Metabolites Detection in Human Sweat. *Anal. Chim. Acta* **2023**, *1278*, 341714.
11. Gillan, L.; Jansson, E. Molecularly Imprinted Polymer on Roll-to-Roll Printed Electrodes as a Single Use Sensor for Monitoring of Cortisol in Sweat. *Flex. Print. Electron.* **2022**, *7*, 025014.
